# Nutritional and Performance Effects of Partial Replacement of Maize With Cassava Root (*Manihot esculenta* Crantz) Meal in Broiler Diets

**DOI:** 10.1002/fsn3.72046

**Published:** 2026-06-28

**Authors:** Shahabuddin Ahmed, Hemayet Hossain, Khadiza Akter Brishty, Aminul Islam, M. Shalim Uddin, Md. Mahfujur Rahman, Mrityunjoy Biswas

**Affiliations:** ^1^ Department of Animal Nutrition, Faculty of Veterinary, Animal and Biomedical Sciences Khulna Agricultural University Khulna Bangladesh; ^2^ Department of Agro Product Processing Technology Jashore University of Science and Technology Jashore Bangladesh; ^3^ Department of Anatomy & Histology Sylhet Agricultural University Sylhet Bangladesh; ^4^ Department of Zoology (GSSC) University of Dhaka Dhaka Bangladesh; ^5^ Poultry Research Center Bangladesh Livestock Research Institute Savar Dhaka Bangladesh; ^6^ Oilseeds Research Center Bangladesh Agricultural Research Institute Joydebpur Gazipur Bangladesh; ^7^ Department of Medicine Sylhet Agricultural University Sylhet Bangladesh

**Keywords:** antioxidant properties, broiler, cassava root meal, feed cost, meat quality, poultry nutrition

## Abstract

The abundance and affordability of cassava root, a carbohydrate‐dense tuber, in tropical regions have drawn attention to it as a possible substitute of conventional energy source in poultry ration. This study investigated the impact of substituting maize meal with varying amounts of cassava root meal on the growth performance, carcass characteristics, hemato‐biochemical parameters, meat composition and sensory properties of broiler chickens. In this study, 400 broiler chicks (Arbor Acres) were assigned to four treatment groups: T_1_ (0%, diet without cassava root meal; CRM) and T_2_, T_3_ and T_4_ containing 10%, 20%, and 30% cassava root meal, respectively. Each group had five replicates consisting of 20 birds each, and trial ran for 35 days. The results indicated that broilers in the T_2_ group had significantly higher body weight, feed intake and feed efficiency ratio, with a lower feed conversion ratio (FCR). T_2_ also showed exceptional carcass characteristics without adversely affecting meat quality and blood parameters of broilers. The proximate composition of breast and thigh muscles showed significant changes (*p* < 0.05). In breast muscle, crude protein (CP), ash, and metabolizable energy (ME) increased with higher cassava root meal (CRM) inclusion, peaking in T_4_. In thigh muscle, crude protein (CP) significantly improved in all CRM‐based diets compared to the control (15.30%), reaching a maximum in T_3_ (20.28%). The diet having 10% cassava root meal (CRM) had the highest amount of total phenolic content, total flavonoid content and DPPH scavenging activity along with the best overall acceptability. The results indicate that cassava root meal can be used as a substitute for maize grains in broiler diets, without any adverse effects on the broiler's growth performance and general health status. It is suggested that maize can be replaced by 10% cassava root meal (CRM) for promoting the growth, meat quality and healthy blood profile of broilers.

## Graphical abstract

Partial replacement of maize with cassava root meal (CRM) improved growth performance, carcass traits, antioxidant activity, meat quality, and sensory acceptability in broiler chickens. Among all treatments, 10% CRM supplementation showed optimal feed efficiency, water‐holding capacity, and antioxidant status without adverse health effects, indicating that moderate CRM inclusion is a sustainable and cost‐effective alternative energy source in poultry diets.

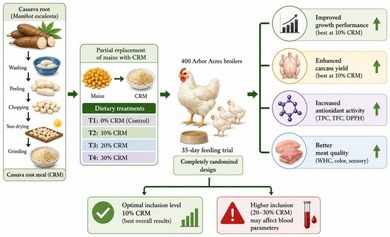

## 1 Introduction

Poultry production is one of the fastest‐growing sectors in the livestock industry worldwide, driven by the increasing demand for high‐quality protein sources such as chicken meat (Choi et al. 2023; Raquib et al. 2022). However, the high and variable cost of conventional feedstuffs (especially maize) present major constraints to sustainable broiler production (Siddik et al. 2026). As the main source of energy in poultry feed, maize represents a large proportion of feed costs and must also contend with human consumption and industrial use (Erenstein et al. 2022). Now‐a‐days it is an alarming issue for the inadequate supply of maize as main energy source feed ingredient for livestock feeding (Ahiwe et al. 2018).

The increasing growth of the animal food production sector and the continuous increase of the global population have caused growing concerns about the challenge for food supplies between humans and animals. This issue holds significant impact, particularly in developing countries like Bangladesh. Therefore, it is vital to evaluate feed alternatives to energy that have the ability to substitute grain (Ahmed et al. 2026). Cassava ( *Manihot esculenta* ) has superior land productivity, with yields ranging from 25 to 60 metric tons per hectare, surpassing those of maize (Erenstein et al. 2022). Furthermore, cassava meal has resulted in reduced manufacturing expenses and has a greater starch content that spans a wider range from 70% to 80% (Chang'a et al. 2020). A study recommended the potentials of using cassava root as a viable substitute for maize in chicken feed (Chang'a et al. 2020). Crude protein and carbohydrates are the main constituents of the cassava root (Abdul Kuddus et al. 2020; Alam et al. 2024; Stupak et al. 2006). Cassava root is rich in carbohydrates, ash, crude protein, and minerals (Ahmed et al. 2024). Moreover, it exhibited 2,2‐diphenyl‐1‐picrylhydrazyl (DPPH) free radical activity (Yi et al. 2011). Because of its elevated starch content, cassava root meal has the capacity to function as a viable substitute for maize and other cereal grains. The application of suitable processing techniques, such as boiling, sun‐drying, marinating, and fermenting, can lower the amounts of cyanogenic glycosides in cassava (Omede et al. 2017).

Numerous research studies of the effects of using pre‐treated or cassava instead of maize meal as an energy source in chicken feed (Alade et al. 2020; Chukwukaelo et al. 2018; Okrathok et al. 2023). They have also looked at how to increase the availability of glucose and improve the efficiency of its absorption (Alade et al. 2020; Chukwukaelo et al. 2018; Okrathok et al. 2023). Previous study has displayed that including cassava root concentrate into broiler meals might yield positive outcomes when the nutrients are appropriately harmonized (Diarra et al. 2015), including potential improvements in the systemic antioxidant status of the broilers due to the presence of bioactive phenolic compounds.

However, there is a lack of comprehensive data regarding the physiological safety of high‐level CRM inclusion and its direct effect on meat oxidative stability. The impact of alternating fed maize to cassava root on growth performance, carcass features, and meat quality of broiler chickens, however, was not extensively investigated. We hypothesize that the partial replacement of maize with CRM will support optimal growth while enhancing the nutritional profile and antioxidant capacity of the meat without compromising the hemato‐biochemical health of the broilers. The purpose of this study was to investigate the effects of replacing maize with cassava root on broiler chicken diets on growth performance, carcass features, hemato‐biochemical profile, breast and thigh meat composition, and sensory attributes. Additionally, this study aimed to quantify the antioxidant potential of CRM‐fed broiler meat using DPPH assays to determine its value as a functional feed ingredient.

## 2 Materials and Methods

### 2.1 Ethics Statement

The Ethical Review Committee of the Jashore University of Science and Technology, Jashore, Bangladesh, provided permission for the animal‐based research methods used in this study (ERC/FBST/JUST/2023‐152).

### 2.2 Preparation of the Cassava Root Meal

Freshly harvested cassava roots from Boda upazilla, Panchagarh District, Bangladesh were cleaned, peeled, and chopped (7–8 cm) into smaller segments (Ahmed et al. 2026). Peeling significantly reduced cyanide content, particularly in sweet cassava varieties where hydrogen cyanide (HCN) is primarily concentrated in the peel. Chopped segments were sun‐dried on protected concrete surfaces for 7 days, reducing soluble cyanide compounds. Sun drying on slanted trays preserved linamarase enzyme activity, facilitating cyanogenic glycoside neutralization (Hassan et al. 2012). To ensure the safety of the ingredient, the processing was conducted until the residual HCN reached safe levels for broiler consumption. Dried cassava chips were ground into a fine powder and stored in polythene bags (Aerni 2006). According to Ahmed et al. (2024) the nutrient values of cassava root meal were as follows: ash 2.96%, crude protein 2.55%, crude fat 0.98%, crude fiber 3.44%, nitrogen free extract (NFE) 82.55% and metabolizable energy (ME) 3061.8 kcal kg^−1^.

### 2.3 Experimental Design, Rations, Treatments and Management of Broiler

In the feeding study, 400‐day‐old commercial broiler chicks (Arbor Acres, AA marketed by Nourish Poultry Hatchery Limited, Dhaka, Bangladesh) of either sex were weighed and randomly allocated to four dietary treatments (T_1_–T_4_) in a Completely Randomized Design (CRD). Each treatment consisted of five replicates with 20 birds per replicate (100 birds per treatment). The treatments included a control diet (T_1_) and diets containing 10% (T_2_), 20% (T_3_), and 30% (T_4_) cassava root meal (CRM) as a partial replacement for maize. The stated inclusion levels of 10%, 20%, and 30% refer specifically to the proportion of maize replaced by cassava root meal (CRM), rather than total dietary inclusion. All experimental diets were formulated on an isocaloric and isonitrogenous basis, where CRM was used as a partial substitute for maize at the respective replacement levels. Growth performance was evaluated weekly, while carcass, hematological, biochemical, meat quality, antioxidant, and sensory analyses were performed at Day 35 using randomly selected birds from each replicate. NRC (1994) standards were followed for creating the broiler ration, which consisted of a starter mash feed for the first 21 days and a grower mash feed for the next 22–35 days. The nutrient contents and trial feed ingredients utilized in this trial are all listed in Table 1. The formulation was performed using (Microsoft Excel Solver) software to ensure a balanced least‐cost ration. The study was conducted in an open‐system chicken house‐oriented east‐to‐west to minimize direct solar radiation. Temperature and relative humidity (RH) were regulated through natural ventilation and manual curtain management, maintaining a brooding temperature of 33°C–35°C in the first week, which was gradually reduced to 24°C by the end of the trial. An appropriate lighting system providing a 23 L: 1 D (23 h light, 1 h dark) photoperiod was implemented to support broiler growth. The broiler chickens were housed in pens on a deep litter floor using rice husks rather than cage systems or individual housing. All birds had ad libitum access to clean drinking water and experimental diets throughout the study period. In accordance with the manufacturer's guidelines (Ceva‐phylaxia Veterinary Biologicals Co. Ltd., Hungary) and recommendations by (Akter et al. 2023), the chicks were immunized against Infectious Bronchitis (IB) using CEVAC BIL, against Infectious Bursal Disease (IBD) using CEVAC IBDL, and Newcastle Disease (ND) using CEVAC NEW L on days (Day, 3, 12, and 21) respectively. Each of the management techniques has been carried out for rearing AA broiler.

**TABLE 1:** Proportion of ingredients used in formulating the experimental diets (as fresh basis).

Treatments: starter (d 0–21); grower (d 21–35)								
Item (%)	T_1_		T_2_		T_3_		T_4_	
	Starter	Grower	Starter	Grower	Starter	Grower	Starter	Grower
Corn	57.00	60.00	51.30	54.00	45.60	48.00	39.90	42.00
Cassava root	—	—	5.70	6.00	11.40	12.00	17.10	18.00
Rice polish	5.00	4.00	5.00	4.00	5.00	4.00	5.00	4.00
Choline chloride	0.10	0.10	0.10	0.10	0.10	0.10	0.10	0.10
Soybean meal	26.00	24.00	26.00	24.00	26.00	24.00	26.00	24.00
Probiozyme	0.05	0.05	0.05	0.05	0.05	0.05	0.05	0.05
Esel dry	0.10	0.10	0.10	0.10	0.10	0.10	0.10	0.10
Fishlog	6.00	6.00	6.00	6.00	6.00	6.00	6.00	6.00
Turbotox	0.10	0.10	0.10	0.10	0.10	0.10	0.10	0.10
Salt	0.25	0.25	0.25	0.25	0.25	0.25	0.25	0.25
NaHCO_3_	0.10	0.10	0.10	0.10	0.10	0.10	0.10	0.10
Methionine	0.20	0.20	0.20	0.20	0.20	0.20	0.20	0.20
Lysine	0.15	0.15	0.15	0.15	0.15	0.15	0.15	0.15
Vitamin premix	0.20	0.20	0.20	0.20	0.20	0.20	0.20	0.20
Sqzyme SME	0.05	0.05	0.05	0.05	0.05	0.05	0.05	0.05
Soybean oil	2.25	2.00	2.25	2.00	2.25	2.00	2.25	2.00
Hamecosaldry	0.20	0.20	0.20	0.20	0.20	0.20	0.20	0.20
Hamecomoltox	0.20	0.20	0.20	0.20	0.20	0.20	0.20	0.20
Lime stone	1.00	1.00	1.00	1.00	1.00	1.00	1.00	1.00
DCP	0.80	1.00	0.80	1.00	0.80	1.00	0.80	1.00
Coccidiostat	0.05	0.05	0.05	0.05	0.05	0.05	0.05	0.05
DB vitamin	0.10	0.10	0.10	0.10	0.10	0.10	0.10	0.10
Toxol powder	0.10	0.10	0.10	0.10	0.10	0.10	0.10	0.10
Total	100	100	100	100	100	100	100	100
*Analyzed values*								
Crude protein %	21.07	20.34	20.70	19.95	20.33	19.57	19.97	19.18
Crude fat %	3.85	3.80	3.83	3.79	3.81	3.77	3.78	3.74
Crude fiber %	3.46	3.37	3.31	3.22	3.17	3.07	3.03	2.92
Calcium %	0.89	0.94	0.91	0.95	0.92	0.96	0.93	0.97
Phosphorus %	0.44	0.47	0.44	0.48	0.45	0.48	0.45	0.49
Methionine %	0.57	0.61	0.56	0.59	0.55	0.59	0.54	0.58
Lysine %	1.24	1.18	1.23	1.17	1.22	1.16	1.22	1.16
*Calculated values*								
ME kcal/kg	3038	3037	3038	3037	3034	3033	3030	3031
Digestible protein %	18.12	17.50	17.68	17.03	17.23	16.56	16.79	16.09

*Note:* Supplied per kilogram of diet—Vitamin A: 12500 IU; vitamin D3: 1250 IU; vitamin E: 18 IU; vitamin K3: 3.7 mg; thiamine: 1.8 mg; riboflavin: 6.6 mg; calcium pantothenate: 10 mg; niacin: 37.5 mg; pyridoxine: 32.5 mg; vitamin B12: 2.5 mg; Mn (manganese sulfate): 50 mg; Zn (zinc sulfate): 37.5 mg; Fe (ferrous sulfate): 25 mg; Cu (tribasic copper chloride): 7.5 mg.

Abbreviations: CRM = cassava root meal, DCP = dicalcium phosphate, Esel Dry = A combination of Vitamin E and Selenium for antioxidant support, Fishlog = A high‐quality fish protein concentrate, Hamecosaldry = A commercial gut acidifier/salinity regulator, ME = metabolizable energy, Rice Polish = A conventional energy and fiber‐rich byproduct of rice milling, Sqzyme SME = A multi‐enzyme complex (Xylanase, Protease, Amylase), T_1_ = control, T_2_ = 10% CRM, T_3_ = 20% CRM, T_4_ = 30% CRM as a partial replacement for maize, Turbotox & Hamecomoltox = multi‐component mycotoxin binders.

### 2.4 Data Collection and Experimental Procedure

#### 2.4.1 Determination of Growth Performance

The initial live weights of the chicks were recorded at the commencement of the investigation using an electronic weighing scale (Model: HESA 3303). Data collection was performed on a weekly basis throughout the experimental period. To determine growth performance, body weights were recorded individually for all birds within each pen, while feed intake was monitored on a per‐pen basis. These primary data points were used to calculate the following parameters following the protocols of Imranuzzaman et al. (2025) and Rahman et al. (2025):

Body weight gain (BWG): BWG = final body weight − Initial body weight

Total feed intake (TFI): TFI = total feed supplied − Feed refusal

$$\begin{array}{c} \text{Feed Conversion Ratio}\;\left(\mathrm{FCR}\right):\mathrm{FCR}=\frac{\text{Total Feed Intake}\;\left(g\right)\;}{\text{Body Weight Gain}\;\left(g\right)}\\ \text{Average Daily Gain}\;\left(\mathrm{ADG}\right):\mathrm{ADG}=\frac{\text{Final weight}-\text{Initial weight}}{\text{Days}}\\ \text{Feed Efficiency Ratio}\;\left(\mathrm{FER}\right):\mathrm{FER}=\frac{\;\text{Body weight gain}\;\left(g\right)}{\mathrm{TFI}}\times 100 \end{array}$$

$$\begin{array}{c} \text{Mortality}\;\left(\%\right):\text{Mortality}=\frac{\text{Number of Birds Died}}{\text{Total number of birds}\;\mathrm{at}\;\text{the beginning}}\times 100 \end{array}$$

#### 2.4.2 Measurement of Carcass Characteristics

At 35 days of age, two broilers (10 from each diet group) were randomly chosen from each replicate, and they were euthanized by cervical disarticulation in order to assess the carcass characteristics, determine the weight of the internal organs, and get the necessary samples. The percentage of the chicken's dressed weight that was made up of its thigh, drumstick, shank, breast muscle, wing, and internal organs (spleen, gizzard, liver, heart, and small intestine) was measured. The organ weights of each bird were measured immediately after slaughtering, and the results were expressed as a percentage of the bird's live weight. Each sample was tested three times, and the mean value was utilized to ascertain the result (Nkukwana et al. 2016). The organs' relative weight was subsequently estimated by utilizing the body weight at the time of slaughter (Martínez et al. 2021).

$$\begin{array}{cc} \text{Relative weight}\;\left(\%\right)\;=\; & \text{Weight of the organ or carcass part}\;\left(g\right)\\ & /\text{Live body weight of the broiler}\;\left(g\right)\times 100 \end{array}$$

Yield percentage was calculated as the proportion of dressed carcass weight relative to live body weight.

#### 2.4.3 Hematological and Serum Biochemical Evaluation

Two broilers from each replicate (10 from each diet group) were randomly selected at the age of 35 days. Hematological characteristics were assessed by collecting blood from the wing veins and placing it in labeled containers that contained ethylene diamine tetra acetic acid (EDTA). In order to quantify serum metabolites, an additional set of blood samples was collected in simple vials. The serum was separated after the blood samples were allowed to coagulate following centrifugation. The method reported by Ogbuewu and Mbajiorgu (2023) and Hossain et al. (2025) was used to measure the hematological parameters, which include PCV, RBC, hemoglobin, WBC, lymphocytes, neutrophils, monocytes, eosinophils, basophils, MCH, MCV, and MCHC. AGAPPE Diagnostics Switzerland GmbH devised the AGGAPE test kits, which were used to quantify a variety of blood biochemical markers (total protein, albumin, globulin, glucose, urea, creatinine, cholesterol, AST, ALT, and ALP). A BA‐88A type semi‐automatic chemistry analyzer from Mindray, Nansha, Shenzhen, China was used to evaluate the biochemical parameters of blood.

#### 2.4.4 Meat Quality Analysis

After the required data on carcass characteristics had been recorded, samples of thigh and breast meat were taken from the same birds used for carcass cut evaluation. The breast and thigh meat samples were processed and placed in hermetically sealed plastic bags, then kept in a sub‐zero freezer at a temperature of −4°C for duration of 5 days. The proximate compositions (Moisture, CP, CF, EE, Ash, NFE, and metabolizable energy) of meat was conducted using the AOAC technique (AOAC 2003). The proximate composition was expressed on a dry matter basis without any adjustments or modifications. The physical meat quality attributes were estimated by following the methods of (Ashim and Prabhat 2020).

Accordingly, a method outlined by Cheng et al. (2017) was used to calculate the drip loss and water holding capacity of flesh from the breast and thigh. The following equation was used to calculate drip loss:

$$\text{Drip loss}\%=\frac{\text{Initial weight of fresh meat}-\text{weight after thawing}\;}{\text{Initial weight of fresh meat}}\times 100$$

The interior temperature of the meat was measured using a digital needle‐tipped thermometer (H 1145, Hanna Instruments, Padova, Italy). The following formula (Vargas‐Ramella et al. 2022) was used to determine the cooking loss:

$$\text{Cooking loss}\%=\frac{\text{Weight of fresh meat}-\text{weight of cooked meat}\;}{\;\text{weight of fresh meat}}\times 100$$

A portable pH meter (Orion model 301; Orion, Beverly, MA, US) with an electrode was used to measure the pH levels of the breast and thigh muscles after the animals were slaughtered.

The DPPH radical scavenging activity of the aqueous supernatant obtained from raw breast and thigh meat was evaluated using the Blois method, as described by Alam et al. 2024, with some modifications. Using an SP‐870 TURNER Barnstead US analyzer, the absorbance of the solution was determined at a wavelength of 517 nm. An equation was used to quantify the DPPH radical scavenging activity, and it is as follows:

$$\begin{array}{c} \text{DPPH radical scavenging activity}\;\left(\%\right)\\ =\;\left(1-\frac{\text{Absorbance of sample}}{\text{Absorbance of control}}\right)\times 100 \end{array}$$

The total phenolics were determined by calorimetry using the Folin–Ciocalteu reagent and, with somewhat modified methods, the methods described by Alam et al. (2024). The measurement of flavonoid contents in the breast and thigh meat extracts was conducted using an updated colorimetric approach described by Sakanaka et al. (2005), with quercetin being used as the reference compound. Each sample was tested three times to make determinations.

#### 2.4.5 Sensory Evaluation

The sensory evaluation protocol for poultry meat was reviewed and institutionally approved by the Ethical Review Committee of Jashore University of Science and Technology, Jashore, Bangladesh (ERC/FBST/JUST/2023‐152). A team of 10 trained panelists was recruited through internal university advertisements. To be eligible, participants had to be self‐reported poultry meat consumers within the age range of 18–65 years. Exclusion criteria included individuals with known food allergies (particularly to poultry or specific seasonings), non‐consumers of poultry, or those under the age of 18. Following the study (Radikara et al. 2016), poultry meat samples (breast and thigh muscles) were collected immediately after slaughter. Following strict food safety protocols, samples were individually vacuum‐packed in food‐grade polyethylene bags and stored at −20°C to prevent microbial growth. For preparation, samples were transported to the sensory laboratory in insulated cold boxes (at 4°C) and boiled without salt or spices until an internal core temperature of 75°Cwas reached, as verified by a digital meat thermometer. Each panelist consumed one sample per treatment in a randomized order. Panelists evaluated color, taste, texture, and flavor using a 9‐point hedonic scale (1 = dislike extremely; 9 = like extremely). To prevent sensory fatigue, water and unsalted crackers were provided as palate cleansers between samples. The collected sensory data were analyzed using STAR (Statistical Tool for Agricultural Research) software (version 2.0.1, 2014) via One‐way Analysis of Variance (ANOVA), with mean separations performed using Tukey's Honestly Significant Difference (HSD) test at a significance level of *p* < 0.05.

### 2.5 Statistical Analysis

Data were subjected to a one‐way Analysis of Variance (ANOVA) using the Statistical Tool for Agricultural Research (STAR, version 2.0.1, 2014) software, developed by the International Rice Research Institute (IRRI). To evaluate the dose–response relationship, orthogonal polynomial contrasts were employed to test for linear and quadratic effects of increasing levels of cassava root meal in the diet. Treatment means were compared using the Least Significant Difference (LSD) test at a 5% significance level (*p* < 0.05). All graphical representations were generated using the ggplot2 package in R Studio (version 4.3.3).

## 3 Results

The analyzed nutrient composition of the experimental diets is presented in Table 1, showing slight variation in CP and ME values among treatments. The rising percentage of CRM correlates with a marginal decline in metabolizable energy. The analyzed nutrient composition of the experimental diets is presented in Table 1. The analyzed CP values ranged from 19.97% to 21.07% in starter diets and 19.18%–20.34% in grower diets, while the calculated ME remained consistent across treatments at approximately 3030–3038 kcal/kg.

### 3.1 Growth Performance

The growth performance of broilers fed diets containing different levels of cassava root meal is presented in Table 2. Initial body weights were similar among all treatments (*p* > 0.05), indicating uniformity at the start. However, significant differences (*p* < 0.05) were observed in final body weight, with the highest weight recorded in T_2_ (2087.94 g), followed by T_3_ (1988.28 g), T_4_ (1957.26 g), and the lowest in control group T_1_ (1901.70 g). A similar trend was noted in average daily gain (ADG), where T_2_ birds showed superior growth (58.37 g/day). Weekly body weights and feed intake were significantly influenced by treatments across all weeks (*p* < 0.05), with T_2_ showing higher values. FCR was the best in T_2_ during Week 5 (1.49), while T1 showed the poorest FCR (1.66). FER followed a similar pattern, being highest in T_2_ across all weeks, particularly Week 1 (140.80%) and Week 5 (67.19%). Mortality rates remained low across all groups but showed slight variations, with T_2_ consistently exhibiting lower mortality throughout the trial (*p* < 0.05). Overall, inclusion of cassava root meal at appropriate levels (T_2_) enhanced broiler growth performance, feed efficiency, and survivability compared to other treatments (Table 2).

**TABLE 2:** Growth performance of experimental broilers diets on graded levels of cassava root meal.

Growth parameters	Treatments				
	T_1_	T_2_	T_3_	T_4_	Significance level
Initial body weight (g)	44.86 ± 0.92	44.76 ± 1.06	44.58 ± 0.89	45.30 ± 1.13	ns
Final body weight (g)	1901.70 ± 0.73^d^	2087.94 ± 0.66^a^	1988.28 ± 0.64^b^	1957.26 ± 0.61^c^	***
Final body weight gain (g)	1856.84	2043.18	1943.70	1911.96	
ADG (g)	53.05	58.37	55.53	54.62	
*Body weight gain (g)*					
1st week	132.26 ± 1.03^d^	164.60 ± 1.43^a^	152.08 ± 0.93^b^	145.34 ± 1.84^c^	***
2nd week	379.08 ± 9.65^d^	454.48 ± 1.17^a^	432.54 ± 0.95^bc^	427.22 ± 2.08^c^	***
3rd week	733.56 ± 3.86^d^	775.88 ± 2.20^a^	760.54 ± 1.61^bc^	750.40 ± 1.90^c^	***
4th week	1304.68 ± 0.47^d^	1397.70 ± 0.70^a^	1376.52 ± 0.85^b^	1361.40 ± 0.65^c^	***
5th week	1901.70 ± 0.73^d^	2087.94 ± 0.66^a^	1988.28 ± 0.64^b^	1957.26 ± 0.61^c^	***
*Total feed intake (g)*					
1st week	106.04 ± 1.24^c^	117.04 ± 1.83^b^	130.64 ± 1.27^a^	130.76 ± 1.47^a^	*
2nd week	420.82 ± 2.25^b^	475.48 ± 1.27^a^	473.02 ± 0.66^a^	471.82 ± 0.91^a^	**
3rd week	930.54 ± 5.29^d^	975.46 ± 1.71^a^	967.40 ± 6.22^bc^	951.10 ± 1.65^c^	**
4th week	1840.02 ± 0.15^d^	1957.52 ± 0.38^b^	1921.38 ± 0.71^c^	1988.14 ± 0.96^a^	***
5th week	3083.30 ± 0.68^d^	3107.64 ± 0.25^a^	3085.72 ± 1.08^b^	3037.18 ± 1.49^c^	***
*Feed conversion ratio (FCR)*					
1st week	0.80 ± 0.004^c^	0.71 ± 0.01^d^	0.86 ± 0.003^b^	0.90 ± 0.01^a^	*
2nd week	1.11 ± 0.003	1.04 ± 0.003	1.09 ± 0.002	1.10 ± 0.006	ns
3rd week	1.26 ± 0.002	1.26 ± 0.003	1.27 ± 0.007	1.26 ± 0.005	ns
4th week	1.41 ± 0.002^b^	1.40 ± 0.003^bc^	1.39 ± 0.004^c^	1.46 ± 0.005^a^	*
5th week	1.62 ± 0.12^a^	1.49 ± 0.005^c^	1.55 ± 0.007^b^	1.55 ± 0.003^b^	*
*Feed efficiency ratio (%)*					
1st week	124.73 ± 0.42^b^	140.63 ± 2.91^a^	116.42 ± 1.36^c^	111.15 ± 0.62^c^	**
2nd week	90.08 ± 0.31^b^	95.58 ± 0.32^a^	91.44 ± 0.20^b^	90.55 ± 0.56^b^	*
3rd week	78.83 ± 0.15	79.54 ± 0.21	78.62 ± 0.42	78.89 ± 0.27	ns
4th week	70.90 ± 0.21^b^	71.40 ± 0.32^a^	71.64 ± 0.45^a^	68.47 ± 0.03^c^	*
5th week	61.67 ± 0.24^b^	67.19 ± 0.53^a^	64.43 ± 0.45^b^	64.43 ± 0.26^b^	*
*Mortality (%)*					
1st week	0.61 ± 0.32^b^	0.56 ± 0.02^ab^	0.54 ± 0.34^a^	0.52 ± 0.23^a^	*
2nd week	0.50 ± 0.01^c^	0.34 ± 0.23^b^	0.36 ± 0.07^a^	0.42 ± 0.09^a^	*
3rd week	0.31 ± 0.05	0.28 ± 0.087	0.28 ± 0.085	0.28 ± 0.34	ns
4th week	0.26 ± 0.03	0.26 ± 0.07	0.24 ± 0.028	0.25 ± 0.05	ns
5th week	0.24 ± 0.04^d^	0.16 ± 0.023^c^	0.19 ± 0.09^b^	0.22 ± 0.078^a^	*

*Note:* 400 broiler hatchlings were randomly assigned to 4 treatments (5 reps/trmt) and fed ad libitum for 35 days varying inclusion levels of CRM. Body weights and feed weights were collected weekly, ^a,b,c,d^Means (± Standard error mean) with different superscript letters in the same rows are significantly different (*p < 0.05, **p < 0.01, ***p < 0.001). The mean values represent 15 birds per treatment group.

Abbreviations: ADG = average daily gain, CRM = cassava root meal, FCR = feed conversion ratio, total feed intake (g)/body weight gain (g), FER = feed efficiency ratio, body weight gain (g)/total feed intake (g) × 100, ns = non‐significant (*p* > 0.05), T_1_ = control, T_2_ = 10% CRM, T_3_ = 20% CRM, T_4_ = 30% CRM as a partial replacement for maize.

### 3.2 Carcass Characteristics

The inclusion of cassava root meal in broiler diets significantly influenced carcass traits and internal organ development (*p* < 0.05) as shown in Table 3. The highest dressing percentage was observed in T_2_ (75.50%), followed by T_3_ (73.12%), with T_1_ and T_4_ showing lower values. Head, neck, thigh, drumstick, breast, abdominal fat, shank, crop, kidney, lung, spleen, liver, heart, and gizzard weights all differed significantly among treatments. Notably, broilers in T_2_ consistently showed superior carcass yields, including highest values for breast (28.21%), drumstick (16.51%), and thigh (15.18%) weights. Internal organ weights such as kidney (1.54%), spleen (0.46%), liver (3.92%), and heart (1.49%) were also highest in T_2_, indicating enhanced physiological development. The lowest values for most parameters were observed in T_1_ and T_4_, suggesting suboptimal utilization at those cassava inclusion levels. Proventriculus weight showed minor variation but remained statistically significant (*p* = 0.031).

**TABLE 3:** Carcass yield characteristics of experimental broilers diets on graded levels of cassava root meal at the age of 35 days.

Parameters	Treatments				
	T_1_	T_2_	T_3_	T_4_	*p*
Dressing weight (%)	72.68 ± 0.52^b^	75.50 ± 0.44^a^	73.12 ± 0.47^b^	72.64 ± 0.41^b^	*
Head weight (%)	2.38 ± 0.02^d^	2.65 ± 0.01^a^	2.58 ± 0.01^b^	2.50 ± 0.01^c^	*
Neck weight (%)	4.35 ± 0.01^c^	4.59 ± 0.04^a^	4.53 ± 0.01^ab^	4.44 ± 0.01^bc^	*
Thigh weight (%)	13.42 ± 0.009^d^	15.18 ± 0.02^a^	14.28 ± 0.02^b^	13.61 ± 0.007^c^	*
Drumstick weight (%)	12.87 ± 0.01^d^	16.51 ± 0.02^a^	14.16 ± 0.02^b^	13.84 ± 0.03^c^	*
Wing weight (%)	11.34 ± 0.009	11.74 ± 0.73	12.33 ± 0.01	11.88 ± 0.02	ns
Breast weight (%)	23.31 ± 0.05^c^	28.21 ± 0.02^a^	25.32 ± 0.06^b^	21.49 ± 0.03^d^	**
Abdominal fat (%)	1.93 ± 0.01^c^	2.37 ± 0.01^a^	2.01 ± 0.007^c^	2.13 ± 0.04^b^	*
Shank weight (%)	3.74 ± 0.01^d^	4.12 ± 0.009^a^	3.95 ± 0.01^b^	3.82 ± 0.008^c^	*
Crop weight (%)	1.15 ± 0.01^d^	1.82 ± 0.02^a^	1.60 ± 0.02^b^	1.23 ± 0.007^c^	**
Kidney weight (%)	1.15 ± 0.01^d^	1.54 ± 0.01^a^	1.33 ± 0.01^b^	1.24 ± 0.01^c^	*
Lung weight (%)	1.33 ± 0.01^d^	1.50 ± 0.02^a^	1.43 ± 0.01^bc^	1.38 ± 0.01^cd^	*
Spleen weight (%)	0.19 ± 0.008^c^	0.46 ± 0.01^a^	0.34 ± 0.01^b^	0.21 ± 0.02^c^	**
Proventriculus weight (%)	0.55 ± 0.01^d^	0.59 ± 0.008^cd^	0.62 ± 0.01^abc^	0.60 ± 0.01^bcd^	*
Liver weight (%)	3.58 ± 0.01^d^	3.92 ± 0.02^a^	3.78 ± 0.01^b^	3.69 ± 0.02^c^	*
Heart weight (%)	1.26 ± 0.008^d^	1.49 ± 0.02^a^	1.41 ± 0.03^ab^	1.35 ± 0.01^cd^	*
Gizzard weight (%)	5.16 ± 0.01^b^	5.60 ± 0.01^a^	5.34 ± 0.01^b^	5.19 ± 0.11b	*

*Note:* The data represent the mean value of 10 samples per treatment; All parameters (except dressing weight) are expressed as relative weights calculated as (Weight of organ or part/live body weight)×100; ^a,b,c,d^Means (± standard error mean) with different superscript letters in the same rows are significantly different (*p < 0.05, **p < 0.01). The mean values represent 15 samples per treatment group.

Abbreviations: CRM = cassava root meal, ns = non‐significant (*p* > 0.05), T_1_ = control, T_2_ = 10% CRM, T_3_ = 20% CRM, T_4_ = 30% CRM as a partial replacement for maize.

### 3.3 Hematological and Biochemical Parameters

Feeding different levels of cassava root meal significantly influenced the hematological and biochemical profiles of broilers at 35 days (*p* < 0.05). Hemoglobin, RBC, PCV, MCH, and MCHC levels were highest in T_1_ and T_2_ but declined significantly in T_4_. WBC count peaked in T_4_ (19,700 × 10^9^/L), indicating a possible stress or immune response. Lymphocyte percentages were highest in T_2_ (73.00%) and lowest in T_3_. Neutrophils were significantly elevated in T_1_ and T_3_. Basophils appeared only in T_4_. Among biochemical parameters, random blood sugar (RBS), uric acid, and BUN levels increased progressively with higher cassava inclusion, with maximum values in T_4_. Cholesterol was lowest in T_2_ and highest in T_4_, while triglycerides were significantly reduced in T_2_. HDL was highest in T_4_ (68.58 mg/dL), and LDL peaked in T_2_. Liver enzymes (AST, ALT, ALP) showed marked variation; ALT and AST were significantly elevated in T_2_, indicating potential hepatic stress. ALP was highest in T_3_ as shown in Table 4.

**TABLE 4:** Hematological and biochemical parameters of broiler on graded levels of experimental diets of cassava root meal at the age of 35 days.

Treatments							
Parameters	T_1_	T_2_	T_3_	T_4_	SEM	Linear	Quadratic
*Hematological parameters*							
Hb g/dl	10.02^a^	9.12^b^	9.26^b^	8.20^c^	0.37	4.24**	0.02
RBC ×10^6^/μL	2.53^a^	2.32^b^	2.23^b^	2.18^c^	0.08	0.19**	0.02
WBC ×10^9^/L	17240^c^	18280^b^	15540^d^	19700^a^	876.22	3229440**	7300800**
PCV %	28.64^a^	27.68^b^	27.70^b^	25.30^c^	0.71	15.00**	1.55**
MCV fL	128.76^b^	130.60^b^	128.80^b^	120.10^a^	2.36	116.04**	82.79**
MCH pg	45.40^a^	43.32^ab^	43.06^b^	39.22^c^	1.29	53.02**	2.32**
MCHC g/dl	35.60^a^	33.34^ab^	34.20^b^	32.72^b^	0.62	9.10**	0.45**
Neutrophil %	32.60^a^	22.80^b^	32.40^a^	25.40^b^	2.48	1310.44**	5.88**
Lymphocytes %	65.00^c^	73.00^a^	62.80^b^	64.40^c^	2.28	21.60**	30.72**
Monocytes %	2.00	2.60	2.60	2.40	0.14	0.22**	0.48**
Eosinophils %	1.60	3.40	2.40	2.60	0.37	0.60**	1.92**
Basophils %	00	00	00	1.08	0.27	1.57**	0.87**
*Biochemical parameters*							
RBS mg/dL	158.62^d^	180.66^c^	203.60^b^	213.44^a^	12.26	5267.81**	111.63**
Uric Acid mg/dL	3.83^d^	4.55^c^	4.86^b^	4.94^a^	0.25	1.99**	0.31**
Total Protein g/dL	2.73^c^	2.95^a^	2.91^a^	2.77^b^	0.05	0.00	0.09**
BUN mg/dl	6.64^c^	7.52^b^	6.67^c^	7.77^a^	0.29	0.94**	0.03**
Cholesterol mg/dL	110.62^b^	100.78^a^	111.56^c^	117.42^d^	3.45	145.73**	184.79**
Triglyceride mg/dL	108.62^a^	80.60^d^	89.64^c^	96.40^b^	5.90	114.42**	907.24**
HDL mg/dL	59.48^c^	45.54^d^	63.58^b^	68.58^a^	4.95	308.35**	269.04**
LDL mg/dL	29.34^c^	39.32^a^	29.92^b^	29.34^c^	2.45	14.12**	78.89**
AST U/I	187.72^b^	208.84^a^	167.18^d^	183.44^c^	8.58	445.54**	17.71**
ALT U/I	9.32^c^	16.30^a^	7.65^d^	9.90^b^	1.90	7.31**	16.85**
ALP U/I	8034^b^	5288^d^	8466^a^	7282^c^	703.71	127512**	1829883**

*Note:* The data represent the mean value of 10 samples per treatment; ^a,b,c,d^Means with different superscript letters in the same rows are significantly different. The mean values represent 15 samples per treatment group.

Abbreviations: ALP U/I = alkaline phosphatase (units per liter), ALT U/I = alanine aminotransferase (units per liter), AST U/I = aspartate aminotransferase (units per liter), BUN mg/dl = blood urea nitrogen (milligrams per deciliter), CRM = cassava root meal, Hb = hemoglobin, HDL mg/dl = high‐density lipoprotein (milligrams per deciliter), LDL mg/dl = law density lipoprotein (milligrams per deciliter), MCH pg. = mean corpuscular hemoglobin (picograms), MCHC g/dl = mean corpuscular hemoglobin concentration (grams per deciliter), MCV fL = mean corpuscular volume (femtoliters), PCV = packed cell volume, RBC = red blood cell, RBS mg/dl = random blood sugar (milligrams per deciliter), SEM = standard error mean, T_1_ = control, T_2_ = 10% CRM, T_3_ = 20% CRM, T_4_ = 30% CRM as a partial replacement for maize, WBC = white blood cell.

** *p* < 0.01.

### 3.4 Proximate Composition of Meat

#### 3.4.1 Breast Muscle Proximate Composition

The proximate composition of breast muscle among the treatment groups is presented in Table 5. Moisture, crude protein (CP), ether extract (EE), crude fiber (CF), and ash contents did not differ significantly among the dietary treatments (*p* > 0.05). However, nitrogen‐free extract (NFE) and metabolizable energy (ME) were significantly affected by cassava root meal (CRM) inclusion (*p* < 0.05). The highest NFE value was observed in T_2_ (1.27%), which differed significantly from the other treatment groups, whereas the lowest value was recorded in T_1_. Similarly, ME content was significantly higher in T_4_ (972.04 kcal/kg) compared to the remaining treatments. Although statistically non‐significant, EE content tended to increase with higher CRM inclusion levels, with the highest value in T_4_ (2.00%) and the lowest in T_2_ (1.03%).

**TABLE 5:** Proximate composition of breast and thigh muscle of broiler on graded levels of experimental diets of cassava root meal at the age of 35 days.

Treatments								
Meat cuts	Parameters	T_1_	T_2_	T_3_	T_4_	SEM	Linear	Quadratic
Breast muscle	Moisture %	75.32	72.78	73.96	72.37	0.66	8.82**	0.68*
	CP %	21.27	22.65	22.98	22.52	0.37	2.49**	2.54**
	EE %	1.80^ab^	1.03^c^	1.60^bc^	2.00^a^	0.21	0.21**	1.03**
	CF%	0.85	0.81	0.50	1.06	0.12	0.02*	0.27**
	Ash %	1.23	1.30	1.30	1.82	0.14	0.47**	0.15**
	NFE %	0.39^ab^	1.27^a^	0.30^b^	0.29	0.24	0.24**	0.59**
	ME (kcal/kg)	894.57^c^	955.67^b^	935.29^ab^	972.04^a^	16.72	6732.91**	438.63**
Thigh muscle	Moisture %	74.05	70.32	72.59	73.21	0.80	0.03	13.69**
	CP %	15.30^c^	18.40^ab^	20.28^a^	18.33^bc^	1.03	18.05**	19.13**
	EE %	1.2	1.7	1.8	1.5	0.13	0.15**	0.48**
	CF%	0.55^d^	1.00^bc^	0.70^c^	1.03^a^	0.12	0.19**	0.01*
	Ash %	0.90^d^	1.01^cd^	2.46^a^	2.04^b^	0.38	3.56**	0.21**
	NFE %	7.99^a^	7.61^b^	4.25^c^	2.07^d^	1.41	67.27**	2.59**
	ME (kcal/kg)	917.18^b^	1055.63^a^	928.02^b^	920.38^b^	33.52	2084.00**	16016.94**

*Note:* The data represent the mean value of 10 samples per treatment; ^a,b,c,d^Means with different superscript letters in the same rows are significantly different. The mean values represent 15 samples per treatment group.

Abbreviations: CF = crude fiber, CP = crude protein, CRM = cassava root meal, EE = ether extract, kcal/kg = kilo calorie/kg, ME = metabolizable energy, NFE = nitrogen free extract, SEM = standard error mean, T_1_ = control, T_2_ = 10% CRM, T_3_ = 20% CRM, T_4_ = 30% CRM as a partial replacement for maize.

* *p* ≤ 0.05.

** *p* ≤ 0.01.

#### 3.4.2 Thigh Muscle Proximate Composition

The proximate composition of thigh muscle showed significant variation in several parameters among the treatments (Table 5). Crude protein (CP) content differed significantly (*p* = 0.027), with the highest value observed in T_3_ (20.28%), followed by T_4_ and T_2_, while the lowest value was recorded in T_1_ (15.30%). Crude fiber (CF) content was significantly higher in T_4_ (1.03%) compared to the other groups (*p* < 0.05). Ash content also varied significantly, with the highest value in T_3_ (2.46%). Nitrogen‐free extract (NFE) decreased significantly with increasing CRM inclusion levels, declining from 7.99% in T_1_ to 2.07% in T_4_% (*p* < 0.05). Metabolizable energy (ME) was significantly higher in T_2_ (1055.63 kcal/kg) than in the other treatment groups.

### 3.5 Meat Quality Attributes

#### 3.5.1 Breast Muscle Quality Attributes

The effects of dietary CRM inclusion on breast muscle quality traits are shown in Table 6. Drip loss (DL) did not differ significantly among the treatments (*p* > 0.05). However, cooking loss (CL) was significantly lower in T_2_ (27.58%) compared to the control group T_1_ (35.95%) (*p* = 0.016). Water‐holding capacity (WHC) was significantly improved in T_2_ (78.22%) relative to all other treatments (*p* = 0.0001). Muscle pH was also significantly affected, with the lowest pH recorded in T_4_ (5.65), differing significantly from the remaining groups (*p* = 0.002).

**TABLE 6:** Quality attributes of breast and thigh muscle of broiler on graded levels of experimental diets of cassava root meal at the age of 35 days.

Meat cuts	Parameters	Treatments					Linear	Quadratic
		T_1_	T_2_	T_3_	T_4_	SEM		
Breast muscle	DL %	10.51	9.19	9.41	9.73	0.29	0.67	2.02
	CL %	35.95^a^	27.58^c^	33.39^ab^	31.68^bc^	1.76	7.35*	33.27**
	WHC %	71.50^c^	78.22^a^	73.16^b^	74.21^ab^	1.43	1.41	24.11**
	pH	6.21^b^	6.12^b^	6.11^b^	5.65^a^	0.13	0.43*	0.10
	DPPH scavenging activity (mg TE/100 g)	44.98^c^	60.62^a^	51.94^b^	51.76^b^	3.20	20.39**	187.70**
	TPC (mg GAE/100 g sample)	32.17^c^	36.40^a^	33.44^ab^	33.19^bc^	0.91	0.00	15.05**
	TFC (mg QE/100 g sample)	27.94^c^	35.49^a^	35.34^ab^	31.31^bc^	1.81	14.88**	100.57**
	L*	63.71^ab^	55.60^bc^	46.50^c^	65.21^a^	4.30	3.17	539.48**
	a*	0.65^b^	0.36^b^	3.80^a^	0.57^b^	0.82	1.54**	6.48**
	b*	10.48^ab^	9.65^bc^	11.39^a^	11.39^a^	0.42	2.99**	0.52
Thigh muscle	DL %	7.15^a^	4.15^d^	5.24^c^	5.73^b^	0.62	1.51**	9.14**
	CL %	36.25^a^	33.44^bc^	32.55^c^	35.36^ab^	0.85	1.90	23.69**
	WHC %	71.55^c^	74.81^a^	72.21^bc^	73.21^ab^	0.71	0.85	3.83
	pH	6.45	6.39	6.33	6.10	0.08	0.18*	0.02
	DPPH scavenging activity (mg TE/100 g)	57.80^b^	73.38^a^	72.29^a^	57.39^b^	4.41	0.79	696.32**
	TPC (mg GAE/100 g sample)	42.17^b^	66.17^a^	41.30^b^	44.08^b^	5.94	54.95**	337.72**
	TFC (mg QE/100 g sample)	44.43^b^	72.35^a^	44.55^b^	48.52^b^	6.70	36.18**	430.20**
	L*	55.24^a^	54.30^a^	48.23^b^	56.38^a^	1.82	1.49**	59.72**
	a*	2.49^a^	2.35^a^	0.98^b^	2.35^b^	0.36	0.48**	1.71**
	b*	9.32^ab^	9.53^a^	4.80^c^	8.24^b^	1.09	9.53**	7.82**

*Note:* The data represent the mean value of 10 samples per treatment. ^a,b,c^Means with different superscript letters in the same rows are significantly different. The mean values represent 15 samples per treatment group.

Abbreviations: a* = greenness to redness, b* = blueness to yellowness, CL = cooking loss, CRM = cassava root meal, DL = drip loss, L* = darkness to lightness, mg GAE/100 g sample = milligram of gallic acid equivalent per 100 g sample, mg QE/100 g sample = milligram of quercetin equivalent per 100 g sample, mg TE/100 g sample = milligram of trolox equivalent per 100 g sample, SEM = standard error mean, T_1_ = control, T_2_ = 10% CRM, T_3_ = 20% CRM, T_4_ = 30% CRM as a partial replacement for maize, TFC = total flavonoid content, TPC = total phenolic content, WHC = water holding capacity.

* *p* ≤ 0.05.

** *p* ≤ 0.01.

Antioxidant‐related parameters were significantly enhanced by CRM supplementation. The highest DPPH scavenging activity, total phenolic content (TPC), and total flavonoid content (TFC) were observed in T_2_, with values of 60.62 mg TE/100 g, 36.40 mg GAE/100 g, and 35.49 mg QE/100 g, respectively, which were significantly higher than those of the other treatment groups (*p* < 0.05).

Regarding meat color attributes, significant differences were observed in L*, a*, and b* values (*p* < 0.05). The highest lightness (L*) and yellowness (b*) values were recorded in T_4_ (65.21 and 11.39, respectively), whereas redness (a*) was significantly higher in T_3_ (3.80) compared to the other treatments.

#### 3.5.2 Thigh Muscle Quality Attributes

The quality traits of thigh muscle were significantly influenced by CRM inclusion levels (Table 6). Drip loss (DL) significantly decreased with increasing CRM supplementation, with the lowest value observed in T_2_ (4.15%) and the highest in the control group T_1_ (7.15%) (*p* = 0.0001). Water‐holding capacity (WHC) was significantly higher in T_2_ (74.81%) compared to the other groups (*p* = 0.039). Cooking loss (CL) was significantly reduced in T_3_ (32.55%) relative to the other treatments (*p* = 0.017). In contrast, pH values did not differ significantly among the treatment groups (*p* = 0.283).

The antioxidant profile of thigh muscle showed marked improvement with CRM supplementation. T_2_ exhibited significantly higher DPPH scavenging activity (73.38 mg TE/100 g), TPC (66.17 mg GAE/100 g), and TFC (72.35 mg QE/100 g) than all other treatment groups (*p* < 0.0001).

Color characteristics also varied significantly among treatments. The highest L* value was recorded in T_4_ (56.38), whereas the lowest was observed in T_3_ (48.23) (*p* = 0.002). Redness (a*) was significantly lower in T_3_ (0.98) compared to the other groups, while yellowness (b*) was also significantly reduced in T_3_ (4.80) (*p* = 0.0001).

### 3.6 Sensory Evaluation of Meat

#### 3.6.1 Breast Muscle Sensory Attributes

**FIGURE 1:**
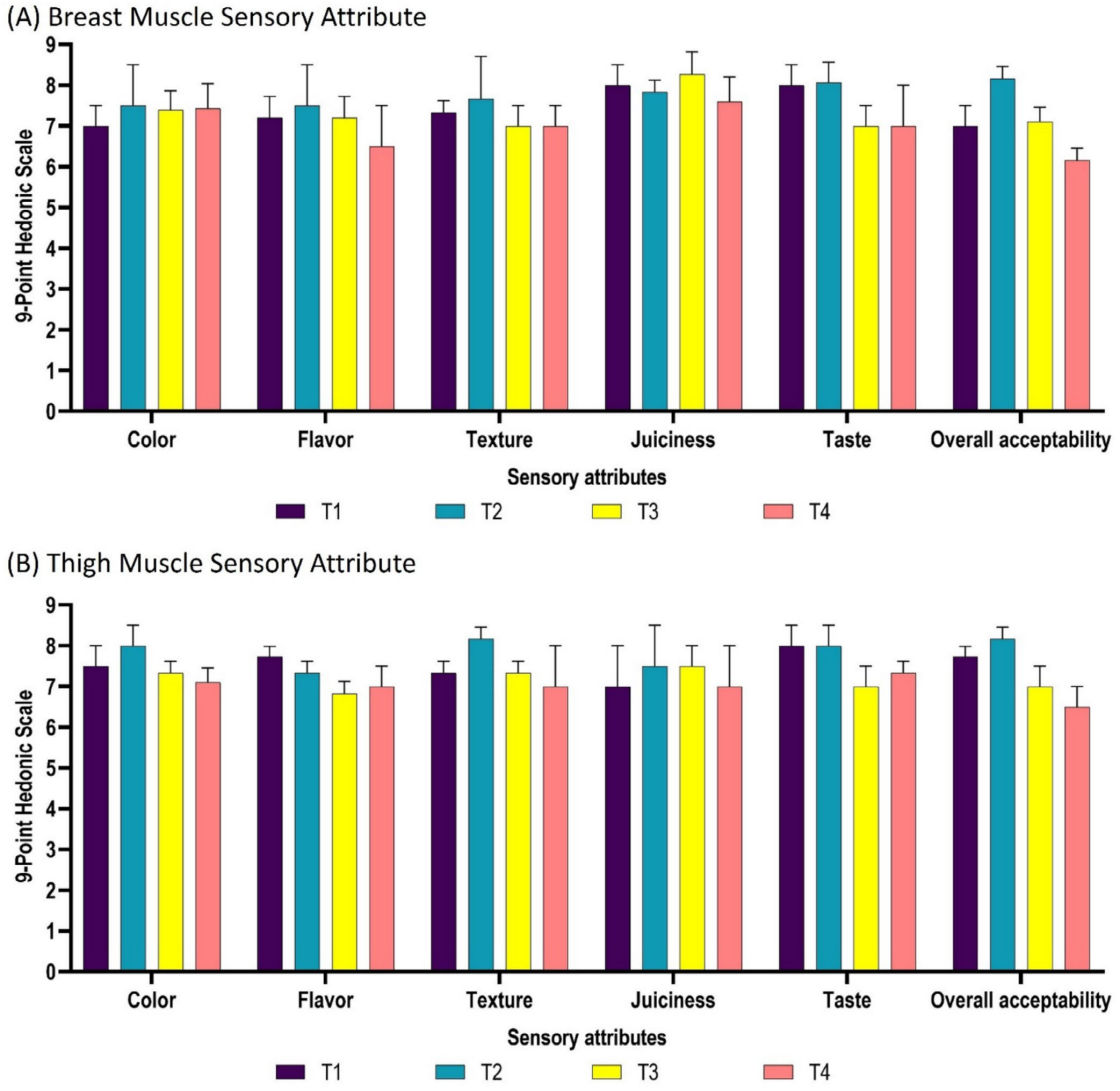
Sensory evaluation of meat/muscle; (A) (breast muscle) and (B) (thigh muscle). (T_1_–T_4_) represents dietary treatments with different inclusion levels of cassava root meal (CRM). T_1_ = control, T_2_ = 10% CRM, T_3_ = 20% CRM, T_4_ = 30% CRM as a partial replacement for maize. Sensory attributes of breast and thigh muscles were evaluated by 10 trained panelists using a 9‐point hedonic scale (1 = dislike extremely, 9 = like extremely). Error bars indicate variability among panelists (± standard deviation/standard error, as applicable). Samples were evaluated for color, flavor, texture, juiciness, taste, and overall acceptability.

The sensory evaluation results of breast muscle are presented in Figure 1. No significant differences were observed among the treatment groups for color, flavor, texture, juiciness, and taste (*p* > 0.05). However, overall acceptability differed significantly among the treatments, with birds fed diets containing up to 10% CRM showing significantly higher overall acceptability scores compared to the control group.

#### 3.6.2 Thigh Muscle Sensory Attributes

In thigh muscle, sensory parameters such as color and juiciness did not vary significantly among the treatments (*p* > 0.05). In contrast, flavor, texture, and overall acceptability showed significant differences (*p* < 0.05), with the control group (T_1_) demonstrating superior scores compared to the CRM‐fed groups. Taste scores did not differ significantly among the treatments, and no CRM treatment group significantly outperformed the control (Figure 1).

## 4 Discussion

The use of cassava root meal (CRM) as an alternative to the conventional energy source maize has demonstrated ameliorative effects on growth performance, biochemical parameters, and meat quality attributes. The cassava root is a popular food source in many African regions and South America for human consumption. It is prepared into various foods, including cassava flour, tapioca, gari, fufu, cassava chips, cassava bread, cassava noodles, and alcoholic beverages (Fathima et al. 2023). It is widely used as an industrial product, similar to bioethanol, biodegradable plastics, textile and paper binders, adhesives, and glues (Fathima et al. 2023; Otekunrin 2024). Unfortunately, its use in livestock or poultry feed is infrequent due to a lack of knowledge on its beneficial effect on poultry as a substitute for maize.

The utilization of innovative feed additives and ingredients to promote broiler chicken production is being investigated on number of trails and the present study specifically focused on including cassava root meal into the diet. When compared to the other treatment groups, birds reared as control group showed a lower body weight and diet having 10% CRM showed higher body weights. The current finding was similar as that of Akapo et al. (2014), who noted that broilers' body weight and feed intake significantly improved when supplementing peeled CRM. A number of studies have shown that giving diets containing between 30 and 35 g kg^−1^ of seaweed meal to chickens has no effect on their ability to get weight (Abudabos et al. 2013; Nhlane et al. 2021). In case of feed intake, the supplementing 10% CRM significantly influence to more feed intake than the other groups. The trend in feed intake similarity across various diets aligns with the findings of Hassan et al. (2012), who documented a notable decrease in feed intake in birds that were fed with more than 25% sun‐dried CRM. Considering FCR, T_2_ group had the lowest FCR throughout the experiment, indicating the most efficient feed utilization. The supplementation of CRM improves FER, suggesting a potential trade‐off between feed intake and feed efficiency at higher levels of cassava root meal. Moderate CRM inclusion may improve growth performance due to its highly digestible starch and readily available energy, which enhance nutrient utilization and protein deposition. However, excessive CRM inclusion may reduce feed efficiency because of lower protein content and residual anti‐nutritional factors.

Nonetheless, this study agrees with the results of Yang et al. (2024), who noted a significant increase in feed consumption in response to a diet's increased cassava meal content during the finisher phase. The findings of other studies reported no significant effects on broiler chickens align with the insignificant results obtained for daily feed intake, FCR, FER, and mortality in broiler chickens fed sun‐dried CRM (Agunbiade et al. 2004; Autónoma De Yucatán et al. 2011; Okpara et al. 2022; Wumnokol et al. 2023).

In broilers, cassava root meal enhances growth performance due to its high carbohydrate content, which serves as a readily available source of energy, contributing to metabolic and body weight gain. Its readily digestible starch helps improve nutrient utilization and feed productivity (Wumnokol et al. 2023). Good nutrient utilization from cassava can be achieved if it is processed correctly, as it contains low levels of anti‐nutritional factors. Additionally, its presence could play a role in maintaining a healthy gut microbiome balance, helping beneficial bacteria and hindering the colonization of pathogens (Akapo et al. 2014). These effects collectively result in a better feed conversion ratio, improved protein synthesis, and increased growth performance in broilers compared to control broilers fed the standard maize diet.

Chukwukaelo et al. (2018) discovered that including cassava meal in the diet resulted in a higher dressing percentage in terms of carcass composition. In this study, we observed that, diet having 10% CRM had the highest dressing percentage. Similarly, Sultana et al. (2012) found that head and thigh weights increased when subjects were fed diets containing 15% and 30% CRM. A study conducted by Abera et al. (2016) revealed an increased proportion of liver in broilers that were fed a diet containing 15% cassava root and leaf meals. Conversely, Morgan and Choct (2016) reported that no notable alterations in yield weight while including 25% cassava meal in the diet. In addition, Abera et al. (2016) and Adeyemo and Longe (2008) observed that broilers fed with 12% cassava meals had higher levels of lungs and kidneys, with the exception of the liver, which showed a more substantial increase when the cassava meal inclusion was at 25%. The high lung weight in broilers is linked to enhanced metabolic activity, which enables sufficient oxygen supply to meet the body's metabolic requirements (Aladi et al. 2021; Namakparvar et al. 2014). Elnour et al. (2020) reported that there were no significant alterations in the weights of breast, abdomen fat, or drumstick but substantial variations in spleen size when cassava root was included in the diet, with an inclusion rate of up to 40%. Abera et al. (2016) confirmed that including 15% cassava in the diet resulted in a higher liver percentage. According to Ukachukwu (2008), liver enlargement and weight gain are associated with detoxifying processes caused by anti‐nutrients. In addition, Alahmari (2024) and Adedokun (2023) linked intestinal expansion to higher levels of indigestible fiber, whereas (Alahmari 2024) attributed the increased weights of the kidneys and heart to the metabolic impact of anti‐nutritional chemicals.

The inclusion of 10% CRM to broiler diets promotes an increase in carcass characteristics, as it enhances the supply of available energy, improves nutrient digestibility, and increases metabolic efficiency, ultimately leading to greater weight and muscle accretion.

Improved carcass yield in T_2_ may be associated with better energy utilization and muscle accretion. Higher CRM inclusion levels may dilute essential amino acids, potentially limiting optimal carcass development. In addition, improved nutrient digestibility may have enhanced protein retention and tissue growth in broilers receiving moderate CRM supplementation.

Differences among studies are attributable to the processing of cassava, levels of inclusion, broiler lines, feeding diets, and ambient conditions (Amos et al. 2021). The existence or depletion of anti‐nutritional factors, such as cyanogenic glycosides and indigestible fiber, also affects organ development and carcass yield, thereby leading to the limited results achieved by different research reports (Adedokun 2023).

Blood indices are essential diagnostic tools for determining the metabolic, health, and nutritional status of an animal. They are employed for the evaluation of the nutritive quality of non‐conventional feedstuffs (Ogbuewu et al. 2017). The inclusion of cassava root in broiler diets resulted in decreased blood hemoglobin (Hb), packed cell volume (PCV), and red blood cell (RBC) values. However, all blood parameters were within the normal range (Hb: 7.0–13.0 g/dL, PCV: 22%–35%, RBC: 2.5–3.5 × 10^6^/μL) (Okafor et al. 2015). The higher MCV in the broilers on a 10% CRM diet implied larger RBCs with normal Hb content. The numbers of WBCs and CKCs, which are important for immunity, were not significantly different, and lymphocytes were within the normal range (51.17%–75.33%) (Kongpechr et al. 2020). Higher root cassava content resulted in decreased MCV and MCH, a sign of both modified RBC size and set‐up, which may increase the risk of anemia. The reduction in erythrocytic indices at higher CRM levels may be linked to residual anti‐nutritional compounds affecting nutrient utilization. Lower cholesterol and triglyceride levels in T_2_ suggest improved lipid metabolism and reduced fat deposition. The maintenance of most hematological values within normal physiological ranges further indicates that moderate CRM inclusion did not adversely affect bird health.

Increased MCH may be suggestive of regeneration anemia (Coles et al. 2008). The concentration of total protein levels, ranging from 3.32 to 4.57 g/dL for birds (Adeyemo and Longe 2008), was unremarkably maintained, denoting that dietary protein was not affected in quality. The broilers fed a diet with 10% CRM (T_2_) showed lower total cholesterol and triglycerides (*p* < 0.05) compared to the control. The activities of AST and ALT were also significantly different between diets and fell within normal reference levels (167.18–208.84 and 7.65–16.30 U/L, respectively), as reported by Coles et al. (2008). Because increased levels of AST, ALT, and ALP can be a sign of liver disease, monitoring these values is essential. Results corroborate Ogbuewu et al. (2017), who associated serum protein status with dietary intake and noted that cassava root meal had no effect on serum proteins and preserved protein quality.

Proximate compositions of broiler breast and thigh muscles showed significant variation due to inclusion of cassava root mix in the diets fed to broilers. According to Chukwukaelo et al. (2018), the highest levels of cassava root mix were reflected in an increase in CP and ME content while decreasing moisture as well as NFE. The CF, EE, and ash content decreased in breast muscle. On the other hand, the CF, EE, and ash percentage in thigh muscle were increased. In this study, we found 10% of CRM improved ME rather than 20% or 30% inclusion of CRM in broiler diet. Furthermore, the present study reveals that the introduction of cassava root mix in broiler diets could cause an evident effect on nutritional attributes and energy content among multiple muscle tissues. Broiler production could be transformed, as crude protein and energy content are improved together with managing other nutritional factors such as moisture and fiber levels according to diet modifications, which will lead to increased broiler meat quality by meeting consumer demand (Chukwukaelo et al. 2018). According to Bogosavljević‐Bošković et al. (2010), the primary elements influencing the chemical composition and quality of poultry meat are the kind of raw materials used and their chemical composition, as well as the ration's protein and energy values. Differences in the nutritional content of meats were seen in the quantities of protein and energy across treatment diets in the current study, and these differences were strongly correlated with the degree of replacement of cassava root meal. Variations in meat composition may reflect altered nutrient partitioning and muscle metabolism due to CRM inclusion. Moderate CRM supplementation improved protein and energy deposition without negatively affecting overall meat quality. Dietary energy balance and nutrient availability may also influence muscle biochemical composition and meat nutritional value.

Meat quality characteristics have a significant impact on consumers' purchasing behavior of products (Mir et al. 2017). Characteristic parameters, such as color, pH, water‐holding capacity (WHC), cooking loss, and juiciness, are important to the meat industry, enabling the production of high‐quality value‐added products (Hussein et al. 2019). Different treatments had a significant influence on the quality characteristics of breast and thigh muscles in this study. Drip loss was significantly decreased in the thigh muscles among the treatment groups, with the T_1_ group demonstrating the lowest value. Cooking loss, however, was significantly (*p* < 0.05) higher in broilers fed the control diet (T_1_). As meat pH has an effect on cooking loss, this is in line with Navid et al. (2010) and Barbut (2015), who stated that pre‐ and post‐slaughter treatments influence the pH of meat. A smaller value in cooking loss represents a more desirable meat quality because it loses less protein during cooking (Abubakar et al. 2021). T_2_ showed the highest levels of total phenolic content (TPC) and total flavonoid content (TFC), as well as the highest antioxidant activity.

Higher antioxidant activity in T_2_ may be associated with phenolic and flavonoid compounds present in cassava, which could improve oxidative stability, water‐holding capacity, and overall meat quality.

The T_4_, T_1_, and T_2_ had the highest L, a, and b* values, respectively, as measured by color. These results are corroborated by the studies of Bakare et al. (2021), who proposed that cassava leaf products (CLP) enhance the stability of meat color and antioxidant capacity. In conclusion, T_2_ (10% CRM) consistently performed the best across all quality parameters.

The results of the study indicated that the sensory attributes of broiler meat were not reduced as a result of the dietary treatments. Abubakar et al. (2021) reported that meat color is affected by muscle composition, structure, condition, and myoglobin type. Organoleptic tests did not reveal differences in meat color among treatments, contrary to (Fofana et al. 2023), who observed that snail meal did not affect the color of meat in broiler. Overall, flavor, which is related to both chemical composition and cooking‐induced changes, was not affected. Texture, with tenderness being a major consumer preference (Warner et al. 2021), was scored as ranging from 7.02 to 8.14 in all groups. Juiciness, a characteristic associated with water‐holding capacity and muscle protein structure (Lorenzo et al. 2019), was also not negatively impacted. The unchanged sensory scores indicate that moderate CRM inclusion did not adversely affect meat palatability, texture, or consumer acceptability. Improved antioxidant stability and water‐holding capacity may also have contributed to maintaining desirable sensory characteristics in CRM‐fed broilers.

Panel differences might indicate variations in dietary habits (Howie et al. 2009). The outcome is similar to that of (Mayulu et al. 2019), who observed that dietary treatments resulted in tastier and consumer‐preferred broiled.

From a poultry nutrition perspective, CRM can serve as a cost‐effective partial substitute for maize when properly processed and nutritionally balanced with adequate protein and amino acid supplementation. However, this study was limited to a single strain and short‐term feeding trial, which may not reflect long‐term health impacts or applicability to other poultry breeds. The present study did not evaluate cyanogenic compounds, nutrient digestibility, gut health, or intestinal morphology, which may provide deeper insight into the nutritional effects of CRM in broilers.

## 5 Conclusion

The dietary inclusion of cassava root meal (CRM) significantly influenced growth performance, carcass traits, hematological and biochemical indices, meat composition, and meat quality attributes of broiler chickens. Among the treatment groups, 10% CRM inclusion (T_2_) produced the most favorable outcomes, including improved body weight gain, feed efficiency, dressing percentage, antioxidant status, water‐holding capacity, and overall meat acceptability without causing detrimental health effects. However, higher inclusion levels negatively affected some hematological and biochemical parameters, indicating possible nutritional imbalance or residual anti‐nutritional effects. Therefore, CRM can be considered a suitable partial substitute for maize in broiler diets at moderate inclusion levels, particularly at 10%, when properly processed and nutritionally balanced. Further studies are recommended to evaluate long‐term effects, nutrient digestibility, and strategies to reduce anti‐nutritional compounds in cassava‐based poultry diets.

## Author Contributions

**Shahabuddin Ahmed:** data curation, investigation, methodology, software, formal analysis, writing – original draft, writing – review and editing. **Hemayet Hossain:** data curation, methodology, software, formal analysis, writing – original draft, writing – review and editing. **Khadiza Akter Brishty:** data curation, methodology, software, formal analysis, writing – original draft, writing – review and editing. **Aminul Islam:** data curation, writing – review and editing. **M. Shalim Uddin:** data curation, writing – review and editing. **Md. Mahfujur Rahman:** data curation, formal analysis, methodology, software, validation, visualization, writing – original draft, writing – review and editing. **Mrityunjoy Biswas:** conceptualization, data curation, investigation, validation, formal analysis, supervision, visualization, resources, writing – original draft, writing – review and editing.

## Funding

The authors have nothing to report.

## Conflicts of Interest

The authors declare no conflicts of interest.

## Data Availability

The data that support the findings of this study are available from the corresponding author upon reasonable request.

## References

[fsn372046-bib-0001] Abdul Kuddus, M. , G. Chandra Datta , M. Mahbubul Alam Miah , et al. 2020. “Performance Study of Selected Orange Fleshed Sweet Potato Varieties in North Eastern Bangladesh.” International Journal of Environment, Agriculture and Biotechnology 5: 673–680. 10.22161/ijeab.53.21.

[fsn372046-bib-0002] Abera, A. , A. Negasi , and U. Mengistu . 2016. “Evaluation of the Carcass Parameters of Growers Fed on Cassava ( *Manihot esculenta* Crantz) Leaf and Root Mixture.” Journal of Biology, Agriculture and Healthcare 6, no. 7: 129–136.

[fsn372046-bib-0003] Abubakar, A. , C. A. Fitri , and H. Koesmara . 2021. “Analysis of pH and Cooking Losses of Chicken Meat due to the Use of Different Percentages of Turmeric Flour.” IOP Conference Series: Earth and Environmental Science 667, no. 1: 012042. 10.1088/1755-1315/667/1/012042.

[fsn372046-bib-0004] Abudabos, A. M. , A. B. Okab , R. S. Aljumaah , E. M. Samara , K. A. Abdoun , and A. A. Al‐Haidary . 2013. “Nutritional Value of Green Seaweed ( *Ulva lactuca* ) for Broiler Chickens.” Italian Journal of Animal Science 12, no. 2: 177–181. 10.4081/IJAS.2013.E28.

[fsn372046-bib-0005] Adedokun, O. O. 2023. “Carcass Characteristics, Organ Proportion, Haematology, Serum Chemistry and Digestibility of Broiler Chickens Fed Umucass 36 Cassava Root Meal.” Fudma Journal of Sciences 7, no. 3: 87–92. 10.33003/FJS-2023-0703-1825.

[fsn372046-bib-0006] Adeyemo, G. O. , and O. G. Longe . 2008. “Effects of Cottonseed Cake‐Based Diets on Performance and Egg Quality Characteristics of Layers.” Pakistan Journal of Nutrition 7, no. 4: 597–602. 10.3923/PJN.2008.597.602.

[fsn372046-bib-0007] Aerni, P. 2006. “Mobilizing Science and Technology for Development: The Case of the Cassava Biotechnology Network (CBN).” AgBioforum 9, no. 1: 1–14.

[fsn372046-bib-0008] Agunbiade, J. A. , B. O. Tolorunji , and H. A. Awojobi . 2004. “Shrimp Waste Meal Supplementation of Cassava Products‐Based Diet Fed to Broiler Chickens.” Nigerian Journal of Animal Production 31, no. 2: 182–188. 10.51791/NJAP.V31I2.1796.

[fsn372046-bib-0009] Ahiwe, E. U. , A. A. Omede , M. B. Abdallh , and P. A. Iji . 2018. “Managing Dietary Energy Intake by Broiler Chickens to Reduce Production Costs and Improve Product Quality.” Animal Husbandry and Nutrition. InTech. 10.5772/INTECHOPEN.76972.

[fsn372046-bib-0010] Ahmed, S. , M. Biswas , A. R. Sunny , et al. 2024. “Nutritional Evaluation of Cassava Meal Components and Maize in Securing Feed and Food.” Integrative Biomedical Research (Former Journal of Angiotherapy) 8, no. 3: 1–7. 10.25163/angiotherapy.839572.

[fsn372046-bib-0011] Ahmed, S. , H. Hossain , M. T. Hossain , et al. 2026. “Cassava Root and Leaf Meal as Alternatives to Energy and Protein Sources in Broiler Diets: Impacts on Growth, Carcass Traits, Blood Biochemistry, Meat Quality.” Open Veterinary Journal 16, no. 1: 560–576. 10.5455/OVJ.2026.v16.i1.53. PMC13314171 42375284

[fsn372046-bib-0012] Akapo, A. O. , A. O. Oso , A. M. Bamgbose , et al. 2014. “Effect of Feeding Cassava ( *Manihot esculenta* Crantz) Root Meal on Growth Performance, Hydrocyanide Intake and Haematological Parameters of Broiler Chicks.” Tropical Animal Health and Production 46, no. 7: 1167–1172. 10.1007/S11250-014-0622-5. 24913764

[fsn372046-bib-0013] Akter, M. S. , M. T. Uddin , and A. R. Dhar . 2023. “Advancing Safe Broiler Farming in Bangladesh: An Investigation of Management Practices, Financial Profitability, and Consumer Perceptions.” Commodities 2, no. 3: 312–328. 10.3390/COMMODITIES2030018.

[fsn372046-bib-0014] Alade, A. A. , A. M. Bamgbose , A. O. Oso , et al. 2020. “Effects of Dietary Inclusion of *Zymomonas mobilis* Degraded Cassava Sifting on Growth Performance, Apparent Nutrient Digestibility, Ileal Digesta Viscosity, and Economy of Feed Conversion of Broiler Chickens.” Tropical Animal Health and Production 52, no. 3: 1413–1423. 10.1007/S11250-019-02146-Z/METRICS. 31782123

[fsn372046-bib-0015] Aladi, N. O. , E. J. Nwafor , V. U. Odoemelam , O. O. Emenalom , I. C. Okoli , and N. J. Okeudo . 2021. “Performance, Carcass, and Organoleptic Scores of Broiler Chickens Fed Diets Containing Wet or Sun‐Dried Fermented Mixture of Grated Cassava Roots and Palm Kernel Cake as Replacements for Maize.” Tropical Animal Health and Production 53, no. 2: 255. 10.1007/S11250-021-02687-2. 33839956

[fsn372046-bib-0016] Alahmari, L. A. 2024. “Dietary Fiber Influence on Overall Health, With an Emphasis on CVD, Diabetes, Obesity, Colon Cancer, and Inflammation.” Frontiers in Nutrition 11: 1510564. 10.3389/FNUT.2024.1510564/BIBTEX. 39734671 PMC11671356

[fsn372046-bib-0017] Alam, M. , M. Jannat , N. Datta , et al. 2024. “The Influence of Microwave‐Assisted Osmotic Dehydration in Coconut Meat Preservation Technique.” Applied Food Research 4, no. 2: 100448. 10.1016/J.AFRES.2024.100448.

[fsn372046-bib-0018] Amos, A. T. , D. U. Kareem , A. O. Amos , and O. M. O. Idowu . 2021. “Nutritional Evaluation of Differently Processed Cassava‐Soya Blends in the Diets of Broiler Chickens.” Nigerian Journal of Animal Production 48, no. 2: 111–127. 10.51791/NJAP.V48I2.2929.

[fsn372046-bib-0070] AOAC International . 2003. Official Methods of Analysis of AOAC International. 17th ed. Association of Official Analytical Chemists (AOAC).

[fsn372046-bib-0019] Ashim, K. B. , and K. M. Prabhat . 2020. “Meat Quality Analysis.” ScienceDirect. 10.1016/C2018-0-01364-X.

[fsn372046-bib-0020] Autónoma De Yucatán, U. , M. Uchegbu , M. C. Etuk , et al. 2011. “Effect of Replacing Maize With Cassava Root Meal and Maize/Sorghum Brewers' Dried Grains on the Performance of Starter Broilers.” Tropical and Subtropical Agroecosystems 14, no. 1: 363–367.

[fsn372046-bib-0021] Bakare, A. G. , P. Cawaki , I. Ledua , et al. 2021. “Quality Evaluation of Breast Meat From Chickens Fed Cassava Leaf Meal‐Based Diets.” Animal Production Science 61, no. 6: 613–619. 10.1071/AN20031.

[fsn372046-bib-0022] Barbut, S. 2015. “Meat Processing, Cooling and Preservation Methods.” In The Science of Poultry and Meat Processing, 313–372. University of Guelph.

[fsn372046-bib-0023] Bogosavljević‐Bošković, S. , Z. Pavlovski , M. D. Petrović , V. Dosković , and S. Rakonjac . 2010. “Broiler Meat Quality: Proteins and Lipids of Muscle Tissue.” African Journal of Biotechnology 9, no. 54: 9177–9182.

[fsn372046-bib-0024] Chang'a, E. P. , M. E. Abdallh , E. U. Ahiwe , et al. 2020. “Replacement Value of Cassava for Maize in Broiler Chicken Diets Supplemented With Enzymes.” Asian‐Australasian Journal of Animal Sciences 33, no. 7: 1126–1137. 10.5713/AJAS.19.0263. 31480161 PMC7322659

[fsn372046-bib-0025] Cheng, Y. , Y. Chen , X. Li , et al. 2017. “Effects of Synbiotic Supplementation on Growth Performance, Carcass Characteristics, Meat Quality and Muscular Antioxidant Capacity and Mineral Contents in Broilers.” Journal of the Science of Food and Agriculture 97, no. 11: 3699–3705. 10.1002/JSFA.8230. 28111775

[fsn372046-bib-0026] Choi, J. , B. Kong , B. C. Bowker , H. Zhuang , and W. K. Kim . 2023. “Nutritional Strategies to Improve Meat Quality and Composition in the Challenging Conditions of Broiler Production: A Review.” Animals 13, no. 8: 1386. 10.3390/ANI13081386. 37106949 PMC10135100

[fsn372046-bib-0027] Chukwukaelo, A. K. , N. O. Aladi , N. J. Okeudo , H. O. Obikaonu , I. P. Ogbuewu , and I. C. Okoli . 2018. “Performance and Meat Quality Characteristics of Broilers Fed Fermented Mixture of Grated Cassava Roots and Palm Kernel Cake as Replacement for Maize.” Tropical Animal Health and Production 50, no. 3: 485–493. 10.1007/S11250-017-1457-7/METRICS. 29098536

[fsn372046-bib-0028] Coles, B. H. , M. Krautwald‐Junghanns , S. E. Orosz , and T. N. Tully . 2008. “Essentials of Avian Medicine and Surgery.” In Essentials of Avian Medicine and Surgery, 3rd ed., 1–397. Wiley. 10.1002/9780470692349.

[fsn372046-bib-0029] Diarra, S. S. , D. Sandakabatu , D. Perera , P. Tabuaciri , and U. Mohammed . 2015. “Growth Performance and Carcass Yield of Broiler Chickens Fed Commercial Finisher and Cassava Copra Meal‐Based Diets.” Journal of Applied Animal Research 43, no. 3: 352–356. 10.1080/09712119.2014.978774.

[fsn372046-bib-0030] Elnour, Z. , M. Babiker , and A. Habib . 2020. “Effect of Different Levels of Cassava Roots on Growth Performance, Carcass Traits and Meat Quality of Broiler Chickens.” International Journal of Livestock Research 10, no. 12: 20–28. 10.5455/IJLR.20200627105321.

[fsn372046-bib-0031] Erenstein, O. , M. Jaleta , K. Sonder , K. Mottaleb , and B. M. Prasanna . 2022. “Global Maize Production, Consumption and Trade: Trends and R&D Implications.” Food Security 14, no. 5: 1295–1319. 10.1007/S12571-022-01288-7.

[fsn372046-bib-0032] Fathima, A. A. , M. Sanitha , L. Tripathi , and S. Muiruri . 2023. “Cassava ( *Manihot esculenta* ) Dual Use for Food and Bioenergy: A Review.” Food and Energy Security 12, no. 1: e380. 10.1002/FES3.380.

[fsn372046-bib-0033] Fofana, D. , A. Ouattara , and M. Diomande . 2023. “Effect of Cashew Meal (Anacardium Occidental) on Organ Yields and Organoleptic Characteristics of Broiler Meat in Côte D'ivoire.” International Journal of Science and Technology Research Archive 4, no. 1: 244–254. 10.53771/IJSTRA.2023.4.1.0045.

[fsn372046-bib-0034] Hassan, A. , M. Tamburawa , C. Alponsus , and J. Yusuf . 2012. “Studies on Growth, Organs Weight and Haematological Parameters of Broiler Chicken Fed Graded Level of Sun‐Dried Cassava Root Meal.” Bayero Journal of Pure and Applied Sciences 5, no. 1: 98–102. 10.4314/BAJOPAS.V5I1.18.

[fsn372046-bib-0035] Hossain, H. , H. Nuradji , M. Y. Miah , M. N. Islam , and M. S. I. Siddiqui . 2025. “Impact of Synbiotic on Growth Performance, Histo‐Architectural Modulation of Lymphoid Organ, Hematology, Blood Biochemistry and Humoral Immune Response in Naked Neck Chicken.” Tropical Animal Health and Production 57, no. 1: 4. 10.1007/S11250-024-04254-X. 39704773

[fsn372046-bib-0036] Howie, J. A. , B. J. Tolkamp , S. Avendano , and I. Kyriazakis . 2009. “The Structure of Feeding Behavior in Commercial Broiler Lines Selected for Different Growth Rates.” Poultry Science 88, no. 6: 1143–1150. 10.3382/PS.2008-00441. 19439622

[fsn372046-bib-0037] Hussein, E. O. S. , G. M. Suliman , A. N. Al‐Owaimer , et al. 2019. “Effects of Stock, Sex, and Muscle Type on Carcass Characteristics and Meat Quality Attributes of Parent Broiler Breeders and Broiler Chickens.” Poultry Science 98, no. 12: 6586–6592. 10.3382/ps/pez464. PMC8913996 31393587

[fsn372046-bib-0038] Imranuzzaman, M. , H. Hossain , F. S. Pory , et al. 2025. “Phytase Supplementation in Broilers: Influence on Growth Performance and Physiological Health.” Journal of Advanced Biotechnology and Experimental Therapeutics 8, no. 2: 342–352. 10.5455/JABET.2025.28.

[fsn372046-bib-0039] Kongpechr, S. , P. Sukon , and D. Sohsuebngarm . 2020. “The Study of Hematology in Commercial Broiler Chickens of Cobb 500, Ross 308, and Arbor Acres Plus.” Journal of Mahanakorn Veterinary Medicine 15, no. 2: 209–221.

[fsn372046-bib-0040] Lorenzo, I. , M. Serra‐Prat , and J. C. Yébenes . 2019. “The Role of Water Homeostasis in Muscle Function and Frailty: A Review.” Nutrients 11, no. 8: 1857. 10.3390/NU11081857. 31405072 PMC6723611

[fsn372046-bib-0041] Martínez, Y. , E. Altamirano , V. Ortega , P. Paz , and M. Valdivié . 2021. “Effect of Age on the Immune and Visceral Organ Weights and Cecal Traits in Modern Broilers.” Animals 11, no. 3: 845. 10.3390/ANI11030845. 33802665 PMC8002570

[fsn372046-bib-0042] Mayulu, H. , A. Rahman , and R. Yusuf . 2019. “Consumer Preference of Broiler Meat Attributes in Traditional Markets.” Hasanuddin Journal of Animal Science (HAJAS) 1, no. 2: 28–36. 10.20956/HAJAS.V1I2.9877.

[fsn372046-bib-0043] Mir, N. A. , A. Rafiq , F. Kumar , V. Singh , and V. Shukla . 2017. “Determinants of Broiler Chicken Meat Quality and Factors Affecting Them: A Review.” Journal of Food Science and Technology 54, no. 10: 2997–3009. 10.1007/S13197-017-2789-Z. 28974784 PMC5603000

[fsn372046-bib-0044] Morgan, N. K. , and M. Choct . 2016. “Cassava: Nutrient Composition and Nutritive Value in Poultry Diets.” Animal Nutrition 2, no. 4: 253–261. 10.1016/J.ANINU.2016.08.010. 29767067 PMC5941045

[fsn372046-bib-0045] Namakparvar, R. , F. Shariatmadari , and S. H. Hossieni . 2014. “Strain and Sex Effects on Ascites Development in Commercial Broiler Chickens.” Iranian Journal of Veterinary Research 15, no. 2: 116–121. 10.22099/IJVR.2014.2349.

[fsn372046-bib-0069] National Research Council . 1994. Nutrient Requirements of Poultry: Ninth Revised Edition. National Academies Press.

[fsn372046-bib-0046] Navid, S. , M. Hilmi , A. Q. Sazili , and A. Sheikhlar . 2010. “Effects of Papaya Leaf Meal, Pineapple Skin Meal and Vitamin D3 Supplementation on Meat Quality of Spent Layer Chicken.” Journal of Animal and Veterinary Advances 9, no. 22: 2873–2876. 10.3923/JAVAA.2010.2873.2876.

[fsn372046-bib-0047] Nhlane, L. T. , C. M. Mnisi , V. Mlambo , and M. J. Madibana . 2021. “Effect of Seaweed‐Containing Diets on Visceral Organ Sizes, Carcass Characteristics, and Meat Quality and Stability of Boschveld Indigenous Hens.” Poultry Science 100, no. 2: 949–956. 10.1016/j.psj.2020.11.038. PMC7858179 33518148

[fsn372046-bib-0048] Nkukwana, T. T. , V. Muchenje , P. J. Masika , E. Pieterse , L. C. Hoffman , and K. Dzama . 2016. “Proximate Composition and Variation in Colour, Drip Loss and pH of Breast Meat From Broilers Supplemented With *Moringa oleifera* Leaf Meal Over Time.” Animal Production Science 56, no. 7: 1208–1216. 10.1071/AN14055.

[fsn372046-bib-0049] Ogbuewu, I. P. , O. O. Emenalom , and I. C. Okoli . 2017. “Alternative Feedstuffs and Their Effects on Blood Chemistry and Haematology of Rabbits and Chickens: A Review.” Comparative Clinical Pathology 26, no. 2: 277–286. 10.1007/S00580-015-2210-0/METRICS.

[fsn372046-bib-0050] Ogbuewu, I. P. , and C. A. Mbajiorgu . 2023. “Dietary *Dialium guineense* Stem‐Bark Supplementation Improves Growth Performance and Haemato‐Biochemical Characteristics of Broiler Chickens.” Heliyon 9, no. 6: e17341. 10.1016/j.heliyon.2023.e17341. 37484235 PMC10361381

[fsn372046-bib-0051] Okafor, B. , G. Kalio , H. Manilla , and O. Wariboko . 2015. “Haematology and Carcass Visual Appraisal of Broiler Chickens Fed Supplemental Diets of Aspilia Africana, Azadirachta Indica and Centrosema Pubescence Leaf Meals in Humid Tropical Nigeria.” American Journal of Experimental Agriculture 7, no. 6: 389–394. 10.9734/AJEA/2015/16152.

[fsn372046-bib-0052] Okpara, O. , O. Obakanurhe , C. N. Onowhakpor , U. G. Sorhue , and O. S. Gbayisomore . 2022. “Effect of Replacing Maize With Processed Cassava on the Growth Performance and Haematological Characteristics of Broiler Chickens.” Animal Nutrition and Feed Technology 22, no. 1: 155–166. 10.5958/0974-181X.2022.00013.0.

[fsn372046-bib-0053] Okrathok, S. , M. Sirisopapong , P. Mermillod , and S. Khempaka . 2023. “Modified Dietary Fiber From Cassava Pulp Affects the Cecal Microbial Population, Short‐Chain Fatty Acid, and Ammonia Production in Broiler Chickens.” Poultry Science 102, no. 1: 102265. 10.1016/J.PSJ.2022.102265. PMC9676379 36402043

[fsn372046-bib-0054] Omede, A. A. , E. U. Ahiwe , Z. Y. Zhu , et al. 2017. “Improving Cassava Quality for Poultry Feeding Through Application of Biotechnology. InTech.” 10.5772/INTECHOPEN.72236.

[fsn372046-bib-0055] Otekunrin, O. A. 2024. “Cassava ( *Manihot esculenta* Crantz): A Global Scientific Footprint—Production, Trade, and Bibliometric Insights.” Discover Agriculture 2, no. 1: 1–20. 10.1007/S44279-024-00121-3.

[fsn372046-bib-0056] Radikara, M. V. , J. C. Moreki , P. M. Kgwatalala , M. H. D. Mareko , and J. B. Machete . 2016. “Meat Quality and Sensory Attributes of Orpington and Indigenous Tswana Chickens Reared Under Intensive System From Day Old to 18 Weeks of Age.” Journal of Animal Science and Veterinary Medicine 1, no. 3: 60–66. 10.31248/JASVM2016.016.

[fsn372046-bib-0057] Rahman, M. , H. Hossain , M. Mia , et al. 2025. “Selection of Efficient Broiler Strain for Productive Performances and Immunity Under Local Farming System in Bangladesh.” Journal of Advanced Biotechnology and Experimental Therapeutics 8, no. 2: 218–231. 10.5455/JABET.2025.18.

[fsn372046-bib-0068] Raquib, A. , A. Uddin , S. M. Nurozzaman , et al. 2022. “Seroprevalence of Mycoplasma Gallisepticum Infection in Layer Chickens of Bangladesh.” Iraqi Journal of Veterinary Sciences 36, no. 1: 9–13. 10.33899/ijvs.2020.127511.1506.

[fsn372046-bib-0058] Sakanaka, S. , Y. Tachibana , and Y. Okada . 2005. “Preparation and Antioxidant Properties of Extracts of Japanese Persimmon Leaf Tea (Kakinoha‐Cha).” Food Chemistry 89, no. 4: 569–575. 10.1016/J.FOODCHEM.2004.03.013.

[fsn372046-bib-0059] Siddik, M. A. , M. A. Khatun , S. R. Mondal , et al. 2026. “Impact of Soybean Meal, Mustard Meal, Rapeseed Meal and Black Cumin on Production Performance, Egg Quality and Gut Microflora of Laying Hens.” Veterinary Medicine and Science 12, no. 2: e70863. 41811204 10.1002/vms3.70863 PMC12978149

[fsn372046-bib-0060] Stupak, M. , H. Vanderschuren , W. Gruissem , and P. Zhang . 2006. “Biotechnological Approaches to Cassava Protein Improvement.” Trends in Food Science & Technology 17, no. 12: 634–641. 10.1016/J.TIFS.2006.06.004.

[fsn372046-bib-0061] Sultana, F. , M. Ali , and I. Jahan . 2012. “Growth Performance Meat Yield and Profitability of Broiler Chickens Fed Diets Incorporating Cassava Tuber Meal.” Journal of Environmental Science and Natural Resources 5, no. 1: 47–53. 10.3329/JESNR.V5I1.11552.

[fsn372046-bib-0062] Ukachukwu, S. 2008. “Chemical Evaluation of *Mucuna cochinchinensis* as Alternative Protein Feeds Stuff.” Journal of Applied Chemistry and Agricultural Research 2, no. 1: 37–41. 10.4314/JACAR.V2I1.41106.

[fsn372046-bib-0063] Vargas‐Ramella, M. , M. Pateiro , D. Rois , et al. 2022. “Effect of Breed and Diet on Carcass Parameters and Meat Quality of Spent Hens.” Annals of Animal Science 22, no. 1: 477–500. 10.2478/AOAS-2021-0036.

[fsn372046-bib-0064] Warner, R. , R. Miller , M. Ha , et al. 2021. “Meat Tenderness: Underlying Mechanisms, Instrumental Measurement, and Sensory Assessment.” Meat and Muscle Biology 4, no. 2: 17–18. 10.22175/MMB.10489.

[fsn372046-bib-0065] Wumnokol, D. P. , S. T. Magaji , S. T. Magaji , et al. 2023. “Performance of Broiler Chickens Fed Graded Level of Sun‐Dried Cassava Root Meal (SDCRM) Based Diets at Starter Phase.” BW Academic Journal 10, no. 1: 5.

[fsn372046-bib-0066] Yang, Y. , F. Lei , Z. Zhang , L. Liu , Q. Li , and A. Guo . 2024. “Effects of Cassava Root Meal on the Growth Performance, Apparent Nutrient Digestibility, Organ and Intestinal Indices, and Slaughter Performance of Yellow‐Feathered Broilers.” Tropical Animal Health and Production 56, no. 8: 274. 10.21203/RS.3.RS-3984805/V1. 39316312

[fsn372046-bib-0067] Yi, B. , L. Hu , W. Mei , et al. 2011. “Antioxidant Phenolic Compounds of Cassava ( *Manihot esculenta* ) From Hainan.” Molecules 16, no. 12: 10157–10167. 10.3390/MOLECULES161210157. 22157579 PMC6264345

